# Therapeutic potential of extracellular vesicles for treating human pregnancy disorders

**DOI:** 10.20517/evcna.2025.07

**Published:** 2025-06-12

**Authors:** Shixuan Zheng, Harry M. Georgiou, Maria I. Kokkinos, Katrina M. Mirabito Colafella, Shaun P. Brennecke, Bill Kalionis

**Affiliations:** ^1^Department of Maternal-Fetal Medicine Pregnancy Research Centre, Royal Women’s Hospital, Parkville 3052, Australia.; ^2^Department of Obstetrics, Gynaecology and Newborn Health, University of Melbourne, Parkville 3052, Australia.; ^3^Department of Physiology, Monash University, Clayton 3800, Australia.; ^4^Cardiovascular Program, Monash Biomedicine Discovery Institute, Monash University, Clayton 3800, Australia.

**Keywords:** Pregnancy, extracellular vesicles, exosomes, therapeutics

## Abstract

Pregnancy complications such as preeclampsia and fetal growth restriction are major global health concerns, contributing to significant maternal and fetal morbidity and mortality. These disorders also increase the long-term risk of cardiovascular, metabolic, and kidney diseases in both mother and child. Accumulating evidence highlights the important role of placental mesenchymal stromal cell (MSC)-derived extracellular vesicles (EVs) in both healthy and pathological pregnancies. In healthy pregnancies, EVs support placental development and maternal-fetal communication. In contrast, EVs from diseased placentas can contribute to pregnancy complications. Importantly, EVs from healthy placental MSCs show promise as novel, cell-free therapies. They offer advantages over whole-cell therapies, including lower immunogenicity, no risk of replication, and easier storage and delivery. This review explores the role of placental MSC-derived EVs in pregnancy disorders, preeclampsia, fetal growth restriction, preterm birth, and gestational diabetes, and discusses their potential as targeted therapeutics. It also examines the future of bioengineered EVs and the challenges that must be addressed to bring EV-based therapies into clinical practice.

## INTRODUCTION

Pregnancy disorders such as preeclampsia (PE) and fetal growth restriction (FGR) are not only common, but also major contributors to fetal and maternal illness and death worldwide. PE alone affects 2%-8% of pregnancies globally, leading to an estimated 46,000 maternal deaths and 500,000 fetal or newborn deaths each year^[1]^. Furthermore, these conditions are associated with an increased risk of developing cardiovascular, metabolic, and kidney diseases in later life for both the mother and child^[2]^. A better understanding of the pathophysiology of these pregnancy disorders is essential for the development of new therapies to improve immediate pregnancy outcomes and prevent long-term health complications for both the mother and child.

Extracellular vesicles (EVs) are key mediators of cell-cell communication throughout development and act via autocrine or paracrine mechanisms to mediate cell signalling^[3]^. EVs, as defined by the International Society of Extracellular Vesicles (ISEV)^[4]^, encompass vesicles of endocytic origin (i.e., exosomes), non-endocytic origin (i.e., microparticles and shed vesicles), and apoptotic bodies^[5]^. The various types of vesicles have different functional properties, but considerable interest has focused on exosomes for their therapeutic potential. EVs contain varying complex cargoes of bioactive molecules, including RNAs, microRNAs (miRNAs), proteins, and lipids^[6]^. EVs are released into the intercellular space, where they mediate proximal cell-cell communication, or they are released into the circulation to mediate distal cell-cell communication. After being taken up by target cells, EVs interact with surface proteins on the target cell and release their cargo into the cellular cytoplasm. This cargo can either repress or initiate a variety of important biological pathways, culminating in functional changes in the target cell^[7,8]^. Importantly, alterations in the microenvironment of the parent cell (i.e., exposure to hypoxia, hormones and growth factors) can modify the EV cargo content and change the concentration of individual components^[7]^.

Increasing evidence reveals that placental multipotent mesenchymal stromal cells (MSCs)-derived EVs are key mediators of both physiological and pathological states in pregnancy^[9,10]^. Research on EVs has revealed their important roles not only in normal placental development but also in the pathophysiology of placenta-associated pregnancy disorders^[11]^. Abnormal EVs isolated from specific placental cell types affected by pregnancy disorders have deleterious effects in many human cell culture and animal disease models^[12]^. Conversely, in an animal model of PE generated by injecting pregnant mice with the sFlt-1 to cause endothelial dysfunction, injection of human umbilical cord MSC-derived exosomes from healthy pregnancies resulted in reduced blood pressure and increased fetal birth weight^[13]^. Furthermore, a human cell culture model of PE was created by treating human umbilical vein endothelial cells (HUVEC) with either the endotoxin lipopolysaccharide (LPS) or PE patient serum to induce endothelial cell dysfunction. Addition of decidual MSC-EVs from healthy pregnancies improved HUVEC dysfunction after either treatment^[14]^. Thus, as described in more detail below, EVs derived from healthy pregnancies have the potential to reverse the detrimental effects of PE.

The placenta from a healthy pregnancy is an abundant, non-invasive and ethically acceptable source of many different types of MSCs, from which EVs can be isolated. MSCs can be obtained from all major placental compartments, and Silini *et al*. have proposed the nomenclature for each type [Table 1]^[15,16]^. Placental MSCs from these different compartments vary in differentiation potential, proliferation rates, and their influence on the immune system. MSC properties exploited in developing therapeutics include low immunogenicity, the ability to migrate and home to diseased or damaged tissue, and the ability to modulate the inflammatory response. Placental MSCs contribute to repairing diseased tissue and wound healing by stimulating angiogenesis, supplying antioxidants, and preventing target cell apoptosis^[17,18]^. These effects are also induced by the EVs they secrete. Furthermore, EVs as therapeutics have significant advantages over the MSCs from which they are derived. EVs do not contain a nucleus and are therefore not capable of replication; they are not cleared by the immune system and have fewer safety, storage, and transport concerns compared with whole MSCs^[19,20]^. Thus, EVs isolated from MSCs are attractive therapeutic agents for treating pregnancy disorders. In the following sections, we explore the role of placental MSC-derived EVs in the pathophysiology of PE, FGR, preterm birth, and gestational diabetes mellitus (GDM), as well as their therapeutic potential for treating these pregnancy disorders and mitigating their long-term health consequences. We also discuss the evidence-based potential for bioengineering EVs to deliver specific cargoes to damaged target cells. Finally, we outline the key barriers to the clinical implementation of EV-based technologies and consider strategies to overcome these challenges.

**Table 1:** MSC types in the human placenta

**Origin**	**Location**	**Source**	**Nomenclature^a^**
FETAL			
	Chorion	Chorion	hCMSC
		Chorionic plate	hCP-MSC
		Chorionic plate MSC-derived from blood vessels	hCP-MSC-bv
		Chorionic villi	hCV-MSC
		Chorion leave	hCL-MSC
	Umbilical cord	Umbilical cord Wharton’s jelly	hUC-WJ-MSC
		Umbilical cord sub-amnion Wharton’s jelly	hUC-saWJ-MSC
		Umbilical cord intermediate Wharton’s jelly	hUC-iWJ-MSC
	Fetal membranes	Amniotic membrane	hAMSC
		Reflected amniotic membrane	hRAMSC
		Placental amniotic membrane	hPAMSC
	Amniotic fluid	Amniotic fluid	hAF-MSC
MATERNAL			
	Decidua	Decidua basalis	hBD-MSC
	Fetal membranes	Decidua parietalis	hPD-MSC

a Recommended nomenclature by Silini *et al*.^[22]^. MSC: Mesenchymal stromal cells.

## PREECLAMPSIA

PE is defined by the International Society for the Study of Hypertension in Pregnancy (ISSHP) as gestational hypertension at or after 20 weeks’ gestation with one or more new-onset disorders of the following types: uteroplacental dysfunction, maternal organ dysfunction (neurological complications, acute kidney injury, haematological complications, liver involvement), or proteinuria^[21]^. PE is termed early-onset PE when diagnosed prior to 34 weeks’ gestation, and late-onset PE when the diagnosis occurs at or after 34 weeks’ gestation^[22]^. Early onset PE is the more severe form of PE and is more often associated with FGR. If PE is not appropriately treated, the patient may develop eclampsia, which involves the onset of seizures^[23]^.

PE occurs in 3%-5% of all pregnancies worldwide and leads to an increased risk of morbidity and mortality in the fetus^[24]^ and mother^[25]^. Despite the prevalence and consequences of PE, the exact aetiology of this syndrome is not fully understood. The theory most widely accepted involves a two-stage process^[26]^. In the first stage, abnormal placentation results in shallow invasion of the maternal spiral arterioles and insufficient remodelling of these vessels. A consequence of insufficient vessel remodelling is hypoxia and subsequent reperfusion injury of the placenta. In the second stage, the placenta releases damaging factors such as reactive oxidative species and cytokines that cause inflammation, oxidative stress, and endothelial cell damage. These damaging factors spread systemically via the maternal circulation, causing injury to the endothelial cells that line the blood vessels. The process culminates in hypertension and organ dysfunction, which manifest as the clinical symptoms of PE^[26,27]^.

Currently, there is no cure for PE other than the removal of the placenta^[28]^. Administration of low-dose aspirin (50-150 mg/daily) is used to prevent PE and improve maternal and fetal outcomes. However, low-dose aspirin treatment is limited to pregnant women who are deemed at risk of developing PE, prior to 16 weeks of gestation^[29,30]^. When symptoms are severe, magnesium sulphate is prescribed for the prevention of seizures^[31]^.

### Abnormal EVs improve our understanding of the pathology of PE

EVs may reflect the immunological status of the mother. When EVs isolated from the peripheral blood of PE patients are incubated with normal peripheral blood mononuclear cells, they stimulate the release of inflammatory cytokines, such as IL-6, indicating an inflammatory role of EVs in PE^[32]^. Additionally, compared to leukocytes from normotensive pregnancies, neutrophils and monocytes from PE pregnancies secrete higher numbers of EVs. In contrast, natural killer cells secrete fewer EVs, reinforcing the concept of reduced immunological tolerance in PE mothers^[33]^. When comparing normotensive and PE pregnancies, placental trophoblast-derived EVs isolated from PE placentae display increased platelet activation^[34]^. Given that platelet activation leads to thrombosis, this suggests that EVs derived from PE placentae contribute to the manifestation of thrombosis, which is a clinical feature in PE patients^[34]^. Moreover, an important placental vasodilator, endothelial nitric oxide synthase (eNOS), is localised on the membrane of trophoblast-derived EVs isolated from plasma of PE pregnancies, and is at reduced levels compared with trophoblast-derived EVs from normotensive controls^[35]^. The vasodilator eNOS maintains endothelial function by synthesising nitric oxide, an antioxidant in endothelial cells, indicating that placental trophoblast-derived EVs from PE pregnancies are associated with the endothelial dysfunction observed in PE^[35]^.

PE is a hypertensive disorder where the change in arterial pressure is a key clinical indicator. A study by Powell *et al*. employed plasma EVs in an *ex vivo* mouse model of the mesenteric artery system. The results showed that EVs derived from the plasma of PE-affected pregnancies increased arterial tone and thereby contributed to the characteristic vasoconstriction of PE, compared to controls^[36]^. These studies suggest that understanding EV properties affected by PE can provide insights into the hypertensive state of PE pregnancies.

### Abnormal EVs and their increased concentration in the maternal circulation are implicated in the pathogenesis of PE

A model of the early stage of PE (i.e., placental hypoxia) was created using EVs isolated from placental trophoblast cells cultured under hypoxic conditions. Dutta *et al*. demonstrated that cultured human umbilical cord endothelial cells (HUVECs) exposed to these hypoxia-derived EVs exhibited increased production of inflammatory cytokines, including VEGF, IL-8, IL-6, and GM-CSF^[37]^. Furthermore, when cultured placental trophoblast cells were incubated with plasma-derived EVs from PE patients, a decrease in cell proliferation and invasion capacity, along with an increase in apoptosis, was observed^[38]^. Additional studies have shown that EVs from PE maternal plasma or PE umbilical cord blood induce dysfunction in various cell types. These include endothelial cells from myometrial arteries, term and early-term trophoblast cells, and HUVECs, which manifested as reduced proliferation, impaired migration, and decreased eNOS production^[39,40]^.

In an *in vivo* human PE model, pregnant rats were injected intravenously with EVs isolated from human placental trophoblast cultured under hypoxic conditions (hypoxic group), or normoxic conditions (normoxic group). The hypoxic group presented with increased blood pressure^[37]^. In another study using a murine PE model, EVs derived from PE placentae were infused into nonpregnant C57 BL/6J female mice, leading to endothelial cell damage, proteinuria, and hypertension^[41]^. These findings provide strong evidence that EVs from women with PE are directly or indirectly involved in the pathogenesis of the disease.

miRNAs are contained within the cargo of EVs and are delivered into the cytoplasm of target cells following their uptake. Following uptake, miRNAs regulate gene transcription of multiple biological pathways in the target cells^[42]^. However, it should be noted that the role of other RNAs in EVs (e.g., long non-coding RNAs), the precise mechanism by which miRNAs are sorted into EVs, and the identity of the specific biological pathways controlled by miRNAs are not fully understood. Thus, further research in this area is required^[43,44]^.

miRNAs delivered by EVs derived from normal placentas exert beneficial effects on target cells by preventing apoptosis and activating repair pathways. In contrast, miRNAs derived from abnormal placental cells are deleterious to target cells^[45]^. For example, miR-199a-5p identified in abnormal EVs isolated from the blood of PE patients contributed to HUVEC dysfunction by inhibiting the expression of SIRT1. Furthermore, EVs that overexpressed miR-199a-5p decreased the production of the vasodilator nitric oxide in HUVECs^[46,47]^. These studies provide further evidence of a direct or indirect role for abnormal EVs and their altered cargo of miRNAs in the pathogenesis of PE.

The availability of multiple animal models of PE^[48]^, along with evidence that abnormal EVs isolated from the circulating blood of PE patients can induce PE-like conditions in cell culture and rodent models, has contributed valuable insights into the pathophysiology of PE. These preclinical models also help to identify and test potential therapeutic targets by assessing the ability of EVs to improve endothelial dysfunction and mitigate the effects of hypoxia. This strategy was supported by a recent study where four antihypertensive drugs (methyldopa, labetalol, nifedipine, and metoprolol), clinically used to treat PE, were tested on HMEC-1 endothelial cells that had been damaged either by phorbol-12-myristate-13-acetate (PMA) or by EVs derived from PE placentae. All four drugs were found to improve endothelial cell function under both conditions^[49]^.

Treatment of pregnant women at high risk of term PE with pravastatin led to a reduction in the concentration of EVs isolated from maternal blood, vasculature, and placental trophoblasts. The STATIN clinical trial showed that the most pronounced effect of pravastatin was the reduction of EVs derived from activated endothelial cells in PE^[50]^. These findings suggest that pharmacological interventions targeting the reduction of abnormal EV concentrations in PE patients may represent a promising therapeutic approach. The observed decrease in EV levels may result from the drug’s effect on EV secretion by parent cells; however, alternative mechanisms cannot be ruled out. It is also possible that changes in EV concentrations are secondary effects of the drug’s action on endothelial or placental trophoblast cells.

#### EV concentrations in maternal blood

EVs are detected in maternal blood as early as 6 weeks of gestation and their concentration increases in the first trimester^[51]^. Salomon *et al*. reported an increased concentration of EVs over gestation in the blood of women with PE compared to normotensive controls^[52]^. In a more recent study, Palma *et al*. developed a rapid and high-sensitivity screening platform to detect maternal plasma concentrations of EVs before 18 weeks of gestation in cases of PE, gestational diabetes, and preterm birth compared to gestation-matched controls. This platform employed EV markers, CD63, CD9, CD81, and TSG101, as well as PLAP. to isolate EVs from maternal plasma. The numbers in the training set for each pathology were 20 or fewer (71 controls), while the test set comprised 8 or fewer (30 controls). The platform identified women at risk of developing a pregnancy disorder with a classification accuracy of > 90%^[53]^.

Whether EV concentration changes in placental disorders, particularly PE, are potential early biomarkers remains unresolved. Popova *et al*. reviewed reports of altered EV concentrations in PE, gestational diabetes, and other pregnancy pathologies^[54]^. A number of these studies suggested that changes in EV numbers may have diagnostic value. However, a recent systematic scoping review of 152 such studies highlighted significant variability and a lack of reproducibility in the methods used to isolate, characterise, and quantify EVs, due to the absence of standardised protocols. Nevertheless, the review concluded that EV numbers generally increase in pregnant women compared with nonpregnant controls, rise with advancing gestation, and are further elevated in certain pregnancy pathologies^[55]^. The authors emphasised the need for further research to clarify gestational changes in EV numbers and to better understand the biological functions of EVs in pregnancy pathologies.

### The therapeutic potential of EVs in PE

A possible therapeutic strategy for PE could be the intravenous injection of MSC-EVs from normal placentas, which may reduce or reverse the harmful effects of EVs secreted by PE-affected placenta tissue.

In a human cell culture model of PE, HUVECs were exposed to damaging stimuli such as LPS or serum from PE patients. The addition of MSC-EVs derived from maternal decidua basalis tissue (which remains attached to the placenta after term delivery in healthy pregnancies - referred to as DMSC-EVs or hBD-MSC) led to enhanced HUVEC proliferation and antioxidant capacity, as well as decreased levels of the pro-inflammatory cytokine IL-6^[14]^. Another HUVEC model of PE revealed that treatment with MSC-EVs from human umbilical cord tissue activated arginine metabolism and increased levels of L-arginine. In an LPS-induced HUVEC PE cell culture model, L-arginine had beneficial effects on multiple aspects of endothelial cell dysfunction^[56]^. These findings suggest that MSC-EVs - particularly those derived from the human umbilical cord or maternal decidua basalis - and L-arginine may be suitable therapeutic targets for PE.

Animal models have reported that EVs isolated from tissues and blood of healthy placentas can alleviate PE-like symptoms. For example, human umbilical cord MSC-EVs injected intraperitoneally into rats with L-NAME-induced PE-like symptoms, including elevated blood pressure and proteinuria, resulted in significant symptom improvement. The therapeutic effects of EVs were attributed to decreased apoptosis and increased angiogenesis in the placenta. A concurrent reduction was also reported in the levels of soluble fms-like tyrosine kinase-1 (sFlt-1), an anti-angiogenic factor elevated in PE patients^[57]^. Another study using a heme oxygenase-1 (HMOX-1) mutant mouse model of PE demonstrated that tail vein injections of Wharton’s jelly MSC-EVs significantly improved PE-like symptoms. This treatment enhanced the remodelling of placental spiral arterioles and led to reductions in blood pressure and proteinuria^[58]^. Yu *et al*. established a PE murine model via tail vein injection of an sFlt-1-overexpressing adenovirus. Treatment of these animals with human umbilical cord MSC-EVs resulted in moderate reductions in hypertension, decreased circulating sFlt-1 levels, and improved kidney function^[56]^.

In addition to ameliorating maternal symptoms, EVs also show the potential in mitigating neonatal complications associated with PE. In the HMOX-1 homozygous mutant PE mouse model, pups exhibited signs of lung dysfunction. However, intravenous administration of human umbilical cord-derived MSCs and MSC-EVs to pregnant PE mice significantly improved pulmonary outcomes in newborns^[59]^.

#### MicroRNA

While MSC-EVs are being investigated as potential therapeutic agents for PE, research on the miRNAs contained in their cargo has shown that these molecules are important regulators of EV function following uptake by target cells^[60]^. For example, human umbilical cord MSC-EVs have been shown to enhance the proliferation and modulate the inflammatory response of placental trophoblast cells via miR-140-5p and miR-195^[61,62]^. In an L-NAME-induced PE rat model, increased levels of miR-18b acted via the Notch2/TIM3/mTORC1 pathway to improve PE outcomes by enhancing trophoblast proliferation and migration while decreasing blood pressure and proteinuria^[63]^. Thus, EVs engineered to overexpress specific, beneficial miRNAs, or suppress harmful ones, hold promise as therapeutic strategies for PE.

The studies described above support the idea that MSC-EVs derived from various placental tissues and maternal blood from healthy pregnancies can improve PE-like symptoms in both *in vitro* and animal models. Moreover, MSC-EV treatments may also offer protective benefits to the fetus against the detrimental effects of PE.

## FETAL GROWTH RESTRICTION

FGR is defined as an estimated fetal weight below the 10th percentile for gestational age^[64]^. It is a leading cause of stillbirth, as well as infant morbidly and mortality worldwide. FGR is defined as early-onset when it occurs before 32 weeks and late-onset after this period^[65]^. Early-onset FGR is often, though not always, associated with PE. The severity of FGR differs between early- and late-onset, with early-onset FGR generally linked to poorer outcomes due to prematurity^[66]^. The underlying causes of FGR may be maternal, fetal, or placental in origin, leading to inadequate nutrient supply for normal fetal growth^[64]^. Maternal risk factors include age, body mass index, and lifestyle behaviours, while fetal risk factors include genomic abnormalities and fetal diseases^[67]^. Known causes of FGR account for up to 40% of cases; the remainder, with no identifiable cause, are classified as idiopathic FGR^[68]^. In FGR, placental lesions are often ascribed to placental ischemia-reperfusion injury. Other forms of placental injuries, such as chorion regression and intervillous thrombi, have also been reported^[69]^. FGR increases the risk of intrauterine fetal demise and neonatal morbidity and mortality^[70]^. Postnatal morbidities in later life include conditions affecting the respiratory, neurological, cardiovascular, and metabolic systems^[71]^. Thus, pre- and postnatal monitoring is recommended for clinical management^[69,72]^.

Current strategies for FGR treatment focus on improving placentation and vascular flow in the placenta during early pregnancy. The use of low-dose aspirin and other antiplatelet agents can prevent or reduce FGR effects at the vascular level, particularly for early-onset PE patients who are at risk of giving birth to FGR-affected fetuses^[73,74]^. Low-dose heparin may also prevent FGR. However, evidence regarding its efficacy remains inconsistent^[74]^.

### EV studies improve our understanding of the pathogenesis of FGR

A porcine model of FGR was used, in which the lightest piglet in each litter was considered FGR-affected (referred to as Intrauterine Growth Restriction, IUGR, in that study), while the heaviest piglets were classified as normal. Lou *et al*. found that normal piglet umbilical cord EVs (NV-EXO) increased HUVEC proliferation *in vitro*. In contrast, FGR-affected umbilical cord EVs (IV-EXO) decreased the proliferation and migration of HUVECs. The pro-angiogenic effects of NV-EXO were attributed, at least in part, to the presence of mIR-150. These data suggest that changes in EVs from FGR-affected piglets are associated with impaired angiogenesis^[75]^.

Another study investigated the effects of maternal alcohol exposure, a known cause of FGR. Tavanasefat *et al*. reported that ethanol treatment in pregnant rats not only affected the protein and RNA content of EVs in adult tissues but also altered the miRNA composition of EVs isolated from amniotic fluid (AF-EVs). Specifically, miR-199a3p, miR-214-3p, and let-7g were significantly increased in AF-EVs following alcohol exposure^[76]^. These studies suggest that alcohol-induced changes in the miRNA cargo of AF-EVs may serve as candidate biomarkers for monitoring alcohol-related FGR.

#### EV concentrations in maternal blood

Ariyakumar *et al*. found that the concentration of EVs in maternal blood was significantly lower in FGR pregnancies compared to healthy controls. Moreover, EVs from FGR cases contained reduced levels of Fas ligand (FasL), a key immune regulatory protein. These observations suggest that EVs may be directly or indirectly associated with inflammatory changes in FGR^[77]^.

### The therapeutic potential of EVs in FGR

Taglauer *et al*. used homozygous HMOX-1 mutant mice as a model of early-onset PE with FGR. Mid-pregnancy HMOX-1 mutant mice showed substantial fetal loss and clear signs of FGR compared to wild-type pregnant mice. Intravenous administration of human umbilical cord Wharton’s jelly MSC-EVs significantly improved FGR-like symptoms, including increased pup weight, reduced inflammation, and enhanced fetal lung function^[58]^. Suboptimal umbilical cord blood function is believed to play an important role in the pathogenesis of FGR. Luo *et al*. isolated piglet umbilical cord vein-derived EVs from piglets with and without FGR. In a cell culture model, EVs from non-FGR piglets substantially improved HUVEC angiogenesis. RNA sequencing of the two EV populations revealed that miR-150 was markedly reduced in EVs derived from FGR piglets. Upregulation of miR-150 promoted angiogenesis in HUVECs, suggesting that miR-150 may regulate angiogenesis and act as both a potential biomarker and therapeutic target for treating FGR in humans^[75]^.

## PRETERM BIRTH

Preterm birth, as defined by the World Health Organisation (WHO), is the delivery of a fetus prior to 37 weeks’ gestation^[78]^. It affects as many as 11%-19% of all live births worldwide^[79]^. Multiple factors such as environmental influences, genetic makeup, and infections can trigger the spontaneous onset of labour^[80-82]^. Management strategies may include the use of tocolytic drugs to prolong pregnancy^[83]^.

Preterm birth is the most common cause of perinatal death among normally formed fetuses. The earlier the gestational age at delivery, the lower the chance of perinatal survival. This condition has severe implications for neonatal health. In the short term, FGR infants are at increased risk of respiratory disorders such as bronchopulmonary dysplasia, as well as neurological conditions including hypoxic-ischemic encephalopathy and cerebral palsy^[84]^. Moreover, preterm infants face a higher risk of long-term morbidities^[85]^.

### EVs in the pathogenesis of preterm birth

Pregnancies complicated by preterm birth are frequently associated with infection and inflammation. Growing evidence implicates EVs in the pathogenesis of preterm birth, particularly in initiating the labour process. For instance, Monsivais *et al*. prepared fetal membrane explants from placentas obtained during term cesarean deliveries not in labour. They also isolated amnion epithelium cells, amnion MSCs, and chorion cells from the same placental tissues. These explants and cell types were treated with or without pro-inflammatory agents (i.e., TNF-α or LPS), after which EVs were isolated and analysed. The protein cargos of EVs from fetal membrane explants differed from those isolated from the individual cell types. While some common proteins were shared, certain proteins were uniquely enriched in the EVs from specific cell types. These findings suggest that EVs derived from fetal membrane tissues and cells respond differently to inflammatory and infectious stimuli. Notably, changes included the upregulation of inflammatory pathways such as IL-6, Rac, and protein kinase signalling, supporting a role for placental EVs in amplifying inflammatory responses during preterm birth^[86]^. Tiozzo *et al*. conducted a similar study using placental explants from normal term deliveries (with or without labour) and from preterm deliveries (< 32 weeks) with or without preterm pre-labour rupture of membranes (PPROM). Upon LPS exposure, EVs were isolated and characterised. Analysis of the conditioned media revealed that LPS treatment decreased TNF-α and increased IL-10 levels - an effect attributed to miR-519c in the EV cargo. These findings suggest that specific miRNAs in placental EVs contribute to placental adaptation to endotoxins and exert anti-inflammatory effects at the maternal-fetal interface, thus playing a role in the pathogenesis of preterm birth^[87]^.

### The therapeutic potential of EVs in preterm birth and fetal development

The potential of EVs as therapeutic agents for complications associated with preterm birth has been previously reviewed^[88]^. More recent studies have emphasised the therapeutic benefits of MSC-derived EVs in treating neonatal lung injuries linked to preterm birth^[89,90]^.

Lee *et al*. used a murine model of hypoxic pulmonary hypertension (HPH), in which hypoxia triggers an inflammatory response in the lungs. EVs were isolated from human umbilical cord Wharton’s jelly-derived EVs (hUC-MSC-EVs) and from human dermal fibroblasts. Treatment with hUC-MSC-EVs, but not human dermal fibroblast EVs, prevented hypoxia-induced signalling that initiates pulmonary inflammation and the subsequent development of pulmonary hypertension affecting the lung epithelium^[91]^. In another study, Willis *et al*. employed a murine model of bronchopulmonary dysplasia, a clinically relevant condition in neonatology. Mice were exposed to hyperoxic conditions and then treated with MSC-EVs before being returned to room air. Untreated mice exhibited abnormal alveolar structures, pulmonary vascular remodelling, and fibrosis. These pathological effects were alleviated by treatment with Wharton’s jelly MSC-EVs, with the underlying mechanism involving modulation of lung macrophage phenotypes^[92]^. Additional studies have reported that intranasal administration of human umbilical cord MSC-EVs in a hypoxic rat model of preterm brain injury reduced neuron cell death, enhanced myelination, and improved learning abilities^[93,94]^.

Specific miRNAs within the EV cargo appear to mediate these therapeutic effects. For example, EVs isolated from placental explants repeatedly exposed to LPS endotoxin - to mimic infection-induced preterm birth - contained increased levels of miRNA-519c, which was found to exert anti-inflammatory effects. miRNA-519c may also serve as a biomarker for placental immune status^[87]^. Similarly, miR-146a-5p and miR-548e-5p, isolated from AF MSC-EVs, have anti-inflammatory effects in LPS-treated HTR8/SVneo human trophoblast cells, partially through modulation of the NF-κB and MAPK signalling pathways. These findings suggest that EV-derived miRNAs could offer promising strategies for treating inflammation-mediated preterm birth^[95]^.

## GESTATIONAL DIABETES MELLITUS

Globally, GDM affects approximately 9% of pregnancies each year and is characterised by the onset of glucose intolerance during pregnancy^[96]^. Depending on the severity of hyperglycaemia, GDM may be managed through dietary modifications or medication. GDM is associated with adverse perinatal outcomes for the mother, including an increased risk of developing Type 2 diabetes and other metabolic disorders later in life^[97]^. For the fetus, risks include being large for gestational age, preterm birth, and an elevated likelihood of developing metabolic syndromes later in life. Thus, postpartum clinical follow-up is recommended for both mothers and neonates^[98]^.

The precise aetiology of GDM remains unclear; however, two common underlying mechanisms are beta-cell dysfunction and tissue insulin resistance, both of which can occur during pregnancy. Beta-cell dysfunction refers to the inability of beta cells to accurately sense blood glucose levels or to secrete sufficient insulin in response^[96]^. Insulin resistance arises when tissues become less responsive to insulin, impairing glucose uptake for metabolism^[99,100]^. In mothers, elevated glucose levels lead to increased oxidative stress and inflammation, contributing to endothelial dysfunction. Excess maternal glucose can also traverse the placental barrier and enter the fetal circulation. In the offspring, this may result in increased adiposity and alterations in brain and nervous system development, potentially affecting behaviour and cognition^[101]^. The first-line treatment for GDM involves dietary modifications and lifestyle changes^[102]^. If these measures prove insufficient, insulin or metformin is prescribed.

### EV studies improve our understanding of GDM pathogenesis

The concentration of secreted EVs is altered in GDM patients. High levels of circulating glucose activate endothelial cells to secrete EVs, which contribute to fetoplacental endothelial dysfunction - an important pathological feature of GDM^[103]^. This indicates that circulating EVs in GDM are abnormal and may contribute to the observed endothelial dysfunction^[104]^.

The number of EVs isolated from the adipose tissue of GDM patients is significantly higher than in healthy pregnancies^[105]^. Proteomic analyses of these EVs have revealed differentially expressed proteins compared with those from adipose tissue in normal glucose-tolerant pregnancies. Pathway analyses showed that these proteins are involved in several dysregulated biological processes, including sirtuin signalling, oxidative phosphorylation, and rapamycin signalling^[106]^. Furthermore, PCR array data demonstrated that GDM adipose tissue-derived EVs upregulate the expression of genes related to glycolysis and gluconeogenesis in placental cells, compared with EVs from normal glucose-tolerant pregnancies^[106]^. These findings suggest that maternal adipose tissue-derived EVs in GDM may adversely affect placental development and contribute, either directly or indirectly, to fetal abnormalities.

EVs isolated from the placental syncytiotrophoblasts of GDM pregnancies exhibited twofold higher dipeptidyl peptidase IV (DPP-IV) activity compared to those from normal glucose-tolerant controls^[107]^. DPP-IV is known to regulate insulin secretion in type 2 diabetes, supporting the notion that GDM and type 2 diabetes share mechanistic similarities at the EV level, and that placental EV cargo contains bioactive molecules that regulate maternal insulin secretion^[107]^. In another study, plasma EVs isolated from women with a history of GDM were incubated with HUVECs to assess endothelial function. These EVs significantly increased the secretion of inflammatory cytokines IL-6, TNF-a, IL-8, and IL-4, compared to EVs from normal glucose-tolerant pregnancies^[108]^.

A human cell culture model further explored this mechanism by comparing EVs isolated from HUVECs incubated with high glucose to those incubated with basal glucose. High-glucose-derived EVs promoted endothelial wound healing and activation, whereas basal-glucose-derived EVs mitigated the effects of high-glucose EVs. These results provide evidence that high glucose induces endothelial dysfunction, at least partly through the secretion of altered EVs^[109]^.

In a rat model of GDM, pre-pregnancy obesity was induced via a high-fat, high-sucrose diet. A positive correlation was observed between elevated endothelial EV concentrations and impaired maternal vascular and cardiac function, compared to lean control rats. EV levels increased more than threefold in the circulation of GDM rats compared to controls and were associated with reduced maternal diastolic function, evidenced by prolonged isovolumetric relaxation time. These findings suggest that elevated EV levels may play a direct or indirect role in vascular damage in GDM^[110]^. In an *in vivo* murine GDM model, nonpregnant mice were infused with plasma-derived EVs from nonpregnant, normal glucose-tolerant, or GDM patients. Mice receiving EVs from GDM patients developed glucose intolerance, whereas those infused with control EVs did not^[111]^.

Bathla *et al*. reviewed changes in microRNA and protein content in EVs affected by GDM, derived from various tissues and fluids. For example, proteins such as GCK, HK3, and PGK2 were upregulated in adipose tissue-derived EVs, while CAMK2b and GLP-1 were increased in plasma-derived EVs^[112]^. Nair *et al*. used a human skeletal muscle cell culture model, stimulated or unstimulated with insulin, and treated with EVs isolated from placental chorionic villous explants of GDM and normal pregnancies. This model simulated abnormal and normal insulin uptake. They found that the concentration of MSC-EVs was increased in GDM pregnancies, accompanied by significant alterations in the miRNA profile. Two EV-associated miRNAs, hsa-miR-125a-3p and hsa-miR-224-5p, were identified, targeting *Glypican 4* and *CD40*, respectively, both of which are implicated in insulin resistance. The study demonstrated that placental EVs contribute to changes in maternal insulin sensitivity in GDM and proposed that placenta-derived EV miRNAs may help regulate skeletal muscle insulin sensitivity during GDM^[113]^.

### The therapeutic potential of EVs in GDM

Placental EVs from normoglycaemic patients have been shown to promote insulin-stimulated glucose uptake and enhance the migration of skeletal muscle cells isolated from patients with GDM^[113]^. Thus, placental EVs may offer therapeutic benefits with regard to insulin sensitivity. In another study, placental MSCs and their derived EVs were isolated from both GDM-affected and healthy pregnant women. It was observed that GDM-MSCs, as well as the EVs they secrete, suppressed the proliferation, migration, and angiogenic capacity of HUVECs. This inhibitory effect was attributed to increased levels of miR-130b-3p^[114]^. When GDM-MSCs were transfected with a miR-130b-3p antagomir, the levels of this miRNA in their EVs were reduced. Consequently, the inhibitory effects of GDM-MSC-EVs on HUVEC proliferation, migration, and angiogenesis were reversed. These findings suggest that inhibiting miRNA-130b-3p in EVs may improve endothelial function in GDM patients^[114]^.

Analyses of EVs in major pregnancy disorders enhance our understanding of disease pathogenesis. Moreover, such studies identify potential therapeutic targets, treatment strategies, and biomarkers. EVs isolated from placental tissues and pregnancy-related fluids affected by these disorders can impair the function of placental cell types from healthy pregnancies, thereby serving as valuable tools for developing human cell culture models. Additionally, administering disease-associated EVs to animals facilitates the creation of *in vivo* models for investigating pregnancy disorders. Furthermore, these EV-based disease models can be used to assess the efficacy of candidate drug therapies. Integrating multi-omics” methods (e.g., RNAseq, proteomics, mass spectroscopy, and lipidomics) with bioinformatics enables the identification of cargo differences between EVs from diseased and healthy pregnancies. These differences reveal candidate miRNAs, proteins, and lipids as potential therapeutic targets or biomarkers. Lastly, EVs isolated from the tissues and fluids of healthy pregnancies may themselves serve as therapeutic agents for treating pregnancy disorders.

## CAN EVs REDUCE THE LONG-TERM CARDIOVASCULAR RISK ASSOCIATED WITH PREGNANCY DISORDERS?

Women who experience pregnancy disorders are at a higher lifetime risk of developing atherosclerosis, heart failure, stroke, chronic kidney disease, and vascular dementia^[2,115-118]^. For instance, those with a history of PE are twice as likely to develop cardiovascular disease (CVD) compared to women with normotensive pregnancies. The risk is particularly elevated in cases of early-onset severe PE, especially when accompanied by recurrent PE or FGR. Women with a history of PE face a two- to four-fold higher likelihood of developing hypertension, the leading risk factor for CVD, within 10-20 years after pregnancy, compared to those who had a normotensive pregnancy^[2,119]^. These findings suggest that although the clinical presentation of PE typically resolves after delivery, residual cardiovascular damage may persist, altering the long-term trajectory of CVD risk in affected women. Similarly, women who experienced GDM are eight times more likely to develop type 2 diabetes than those who did not experience GDM.

We hypothesise that EVs may help alleviate the maternal symptoms of pregnancy disorders by repairing damage to the maternal cardiovascular system, thereby potentially resetting the long-term risk trajectory for CVD. Importantly, two therapeutic windows may exist: during pregnancy and in the early postpartum period^[120]^. Although the clinical manifestations of pregnancy disorders often dissipate following delivery, the underlying tissue damage may take longer to resolve. Mounting evidence, particularly in the case of PE, suggests that microvascular dysfunction can persist^[121,122]^, with reported alterations in both the renin-angiotensin and endothelin systems. Preclinical studies support this hypothesis. In one study, EVs derived from the conditioned medium of term placental explants from normotensive pregnancies were injected into spontaneously hypertensive rats (SHRs) at 3 months of age. The animals were monitored until 15 months of age. SHRs treated with these EVs displayed reduced blood pressure and improved cardiac function^[123]^. These findings highlight the need for future clinical studies to explore the potential of EV-based therapies administered during pregnancy or the early postpartum period to improve long-term maternal cardiovascular outcomes.

## ARE BIOENGINEERED EVs THE NEXT STEP IN TREATING PREGNANCY DISORDERS?

Current bioengineering research focuses on modifying specific components of EVs and adapting them as drug-delivery vehicles^[124,125]^. General strategies for EV bioengineering include genetic or environmental modification of parent cells, loading EVs with therapeutic agents (e.g., biologics or drugs), and modifying or camouflaging the EV membrane. Examples of these approaches include altering the growth environment of parent cells during cell culture (e.g., changing oxygen levels)^[37,126]^, culturing cells on enhanced growth surfaces^[127-129]^, and labelling EVs to enable *in vivo* tracking. Other strategies involve modulating EV function with miRNAs or plasmid DNA via electroporation or transfection agents, targeting EVs to specific tissues or organs, or modifying their uptake by recipient cells^[126]^. For example, in a murine model study by Luo *et al*., EVs from MSCs were conjugated with a bone marrow MSC-targeting aptamer (i.e., an oligomer of artificial ssDNA, RNA, or peptide that binds specifically to a target molecule) to enhance EV accumulation in bone tissue affected by osteoporosis^[130]^. In a breast cancer study, EVs were engineered to express the urokinase plasminogen activator receptor for improved tumour targeting and were used as nanocarriers for miRNAs (antisense-miRNA-21 or antisense-miRNA-10b) in tumour-bearing nude mice. This approach showed evidence of tumour regression and prolonged progression-free survival^[131]^.

In the context of pregnancy, preterm birth was delayed in LPS-induced mice after intraperitoneal injection of EVs engineered to deliver NF-κB inhibitors^[132]^. Another example is a recent study by Kammala *et al*., in which EVs were loaded with recombinant IL-10 via electroporation. When injected into pregnant mice, these EVs delayed preterm birth triggered by *E. coli* infection^[133]^. Recent advances have employed the fusion of EVs with synthetic drug vectors to create hybrid EVs. These hybrids have attracted considerable interest due to their ability to combine the natural tissue-targeting and immunomodulatory properties of EVs with the enhanced drug delivery capabilities of synthetic vectors^[134]^. However, the application of hybrid EVs in human pregnancy disorders remains untested.

## BARRIERS TO USING EVs AS THERAPEUTICS

Significant barriers currently limit the therapeutic application of EVs for pregnancy disorders (see graphical abstract). Below, we outline the key challenges and potential strategies to overcome them.

### EV isolation and characterisation methods

The choice of parental cells for EV production is a major barrier to clinical translation. MSCs, commonly used for this purpose, are present in low numbers in tissues, and there is considerable patient-to-patient variability that affects the composition and cargo of the resulting EVs. Achieving consistent EV composition and cargo across batches, with minimal lot-to-lot variation, remains a significant challenge^[135]^. One potential solution is to use genetically modified, long-term (or immortalised) MSC lines, which exhibit reproducible characteristics and can be expanded over multiple generations for large-scale production^[136,137]^. Although immortalised cell lines can reduce inter-patient variability, other cell culture factors must also be optimised. The culture medium, environmental conditions, and multiple passages during cell expansion can all influence EV content^[138]^. For example, factors such as hypoxia and oxidative stress significantly affect EV composition and secretion from MSCs^[7]^. Additional barriers include contamination with non-EV components, EV loss during isolation, long processing times, and low yields. Challenges also arise in scaling up EV production and preserving EV functionality under thawing conditions^[139]^. These issues may be mitigated by following the MISEV (Minimal Information for Studies of Extracellular Vesicles) protocols described below.

EVs derived from placental tissues (e.g., chorion, decidua, umbilical cord, and Wharton’s jelly) are typically isolated through enzymatic digestion^[140]^, followed by purification from conditioned media^[141]^. In contrast, EVs from biological fluids (e.g., urine, plasma, serum, cord blood, and AF) are isolated either by collecting the total EV population or by targeting placenta-specific EVs using biomarkers. Despite the availability of several conventional EV isolation methods, there is no consensus on a single most effective technique. Ultracentrifugation is widely regarded as the “gold standard” for EV isolation from pregnancy-related samples. However, other methods can be used alone or in combination^[142]^, including ultrafiltration, immunomagnetic bead capture, and size-exclusion chromatography. These alternatives can offer distinct advantages depending on the EV subtype being targeted. Different isolation approaches are often needed for specific EV subpopulations (e.g., exosomes, microvesicles, apoptotic vesicles) due to differences in size, morphology, and surface markers. Each method has unique pros and cons, affecting reproducibility, efficiency, and functional results^[143]^. Advantages of some methods can include ease of use, suitability for large sample volumes, subtype specificity, low costs, scalability, and high purity. Disadvantages may include expensive equipment, contamination risks, time-consuming protocols, low purity, low yield, and limited scalability for clinical applications.

Numerous techniques are employed for EV detection and characterisation, including transmission electron microscopy (TEM), scanning electron microscopy (SEM), cryogenic electron microscopy (cryo-EM), nanoparticle tracking analysis, field flow fractionation, atomic force microscopy, resistive pulse sensing, flow cytometry, and Western blotting^[144]^. Each method offers specific strengths and limitations. Effective protocols typically require only small sample amounts, allow rapid detection, and offer high sensitivity or throughput. However, drawbacks may include high equipment costs, background noise, susceptibility to contamination, and time-consuming procedures with limited sensitivity.

The ISEV released an updated version of the MISEV guidelines in 2023^[145]^. These recommendations provide standardised protocols for EV isolation and characterisation, including from pathological placentae, and highlight the advantages and disadvantages of each method. Most importantly, the MISEV offers standardised reporting criteria to enhance reproducibility and quality in future research publications”.

Technical innovations in EV preparation are advancing rapidly. As noted, the current MISEV guidelines aim to standardise EV isolation, purification, and characterisation. Emerging technologies include microfluidic chip-based platforms and devices integrated with nanomaterials, which improve EV isolation. DNA-based techniques, such as DNA hydrogels, and membrane-based methods are also being developed. Wang *et al*. recently provided a comprehensive review of these state-of-the-art approaches^[146]^. A persistent challenge is that existing technologies do not yield homogeneous EV populations, either across subtypes (e.g., exosomes, microvesicles, and apoptotic bodies) or within subtypes (e.g., exosomes)^[147]^. Advanced tools, such as high-resolution atomic force microscopy with resonance-enhanced infrared spectroscopy, enable detailed analysis of entire EV populations, subpopulations, and individual MSC-EVs^[148]^. Novel flow cytometry techniques also enable the characterisation of both EV populations and single EVs^[149]^. Additionally, high-throughput mass spectrometry and RNAseq are used to analyse the protein, lipid, and RNA cargo of placental MSC-EVs^[137]^.

Liquid biopsy approaches, which screen maternal blood early in pregnancy for placental disorders before clinical symptoms appear, offer the potential for early intervention using EV-based therapeutics. Microfluidics-based liquid biopsy platforms isolated EVs based on physical properties (e.g., size, density, electrical characteristics) or via affinity-based capture using specific probes^[150]^. Nguyen *et al*. reviewed candidate biomarkers for placental disorders that could be used to capture placenta-derived EVs^[151]^. Micro- and nanodevices for lipid biopsy are rapidly evolving in cancer research^[152]^, and their adaptation to detect placental pathologies holds considerable promise.

Human therapies using MSC-EVs require relatively large doses, with effective ranges reported between 0.001 and 100 mg of EV protein per kg of body weight^[153]^. Large-scale production of parental MSCs and their EVs for clinical trials poses another major challenge. Syromiatnikova *et al*. summarised various methods for large-scale EV production^[154]^. Strategies include the use of bioreactors, which may be coated with novel surface chemistries that stimulate MSC proliferation^[129]^. Modifying culture conditions (e.g., hypoxia, serum starvation) can further stimulate both MSC growth and EV secretion. Chemical or physical stressors also boost EV production. Additionally, mechanical disruption techniques, such as sequential filtration through decreasing pore sizes, have been shown to significantly enhance EV yield^[154]^.

### Limitations of cell culture and animal models

Cell culture and animal models are extensively used in preclinical research to generate data that guide clinical trials. However, this poses a particular challenge for developing therapeutics for PE, as the disorder is unique to humans and higher primates^[155]^.

Cell culture models - where EVs are used to treat normal or pathological placental tissues, primary cells, or genetically modified long-term or immortalised cell lines - often inform subsequent animal studies of pregnancy disorders. While these models can capture certain physiological or pathological aspects of the placenta, they fail to fully replicate the complexities of *in vivo* processes, such as extracellular matrix influences and intercellular interactions. Moreover, these studies typically involve only one or a few cell types, whereas native tissues comprise multiple interacting cell populations. Practical limitations include artificial culture conditions that do not mimic the tissue microenvironment, the challenge of maintaining sterility, and phenotypic or genetic drift with repeated cell passaging.

Animal models of PE and FGR are commonly established via surgical intervention, exposure to harmful agents, or by targeting a single biological pathway. Although these models are valuable, PE and FGR in humans are multifactorial disorders^[155]^. There are also significant differences in placental development and morphology between commonly used rodent models and humans. These differences include litter size, neonatal weight, gestation length, the type of placental barrier (haemomonochorial in humans *vs.* haemotrichorial in rodents), the architecture of the maternal-fetal interface (villi in humans *vs.* labyrinth in rodents), and variations in placental steroidogenesis^[156]^. Larger animals, such as sheep, offer models that more closely resemble human pregnancy^[157]^. Other animal models - including the spiny mouse, guinea pig, mouse lemur, and common marmoset - have also shown promise, though they remain underutilised^[158]^. Despite the advantages of murine models - particularly their amenability to genetic manipulation via embryonic stem cell-based knockouts or transgenic approaches - they lack certain physiological parallels with human pregnancy. Nevertheless, researchers benefit from the wealth of biological pathway data available from mouse studies, supported by extensive “omics” databases^[159]^.

In rodent studies where EVs were used to treat pregnancy disorders, dosage varied from a daily dose of 10 to 50 mg/kg of EV protein^[93,94]^. Other studies reported EV doses in absolute amounts (e.g., 10 μg/day) without referencing animal weight^[37]^, highlighting the need for more standardised and refined experimental designs. Moreover, EV treatment in these models typically coincides with the induction of the placental disorder. While informative, the applicability of these findings to humans is limited. In clinical settings, EV administration would likely occur after clinical diagnosis or during disease progression due to the absence of reliable early biomarkers, potentially reducing therapeutic effectiveness. Human preclinical research and clinical trials must address critical variables, including EV safety, required dosage volumes, quality control, dosing schedules, and frequency^[153]^.

To address these translational challenges, Menon *et al*. recently proposed the adoption of “New Approach Methods (NAMs)” as alternatives to traditional human cell culture and small animal models. These methods rely on “humanised models”^[160]^, such as organ-on-a-chip platforms, which simulate pathological conditions. These microfluidic devices replicate key maternal-fetal interfaces (i.e., the fetal membranes and placental trophoblast) or the maternal *decidua basalis*. EVs, whether used as therapeutic agents or drug delivery vehicles, are tested within these systems. Artificial intelligence (AI) plays a complementary role in these efforts - analysing data from humanised models and integrating it with findings from organ-on-a-chip experiments, preclinical studies, and clinical trials. The ultimate goal is to develop comprehensive, predictive models of pregnancy-related pathologies to inform more rational and effective clinical trial designs.

### Targeting the placenta

Another important consideration is how to ensure that EVs specifically target the placenta during treatment. Tong *et al*. demonstrated that intravenous injection of fluorescently labelled human first-trimester EVs into pregnant CD1 mice resulted in EV accumulation in the lungs, kidneys, and liver, as well as uptake by the endothelium. Signals in these organs were detectable as early as 30 min after injection and remained strong after 24 h. However, EV signals in the placenta were barely detectable at either time point^[161]^. Therefore, therapeutic applications of EVs may require strategies to enhance placental targeting to avoid off-target effects on major maternal organs and potentially on fetal organogenesis.

Various strategies have been proposed and tested to direct therapeutics to the placenta or uteroplacental vasculature^[12,162-164]^. These include delivery systems using adenoviral vectors carrying VEGF or IGF-1, liposomes coated with tumour-homing peptides and IGF-2, placenta-homing peptides delivering specific miRNAs, and short interfering RNAs targeting anti-angiogenic, placental-specific sFLT1 mRNA isoforms. These approaches have improved placental and/or fetal function to varying degrees in human cell culture systems and in rodent or sheep models. However, some strategies involve direct injection into the placenta or uterine vasculature, or the use of prolonged subcutaneous infusion via osmotic pumps. Moreover, the translation of these therapies to humans or nonhuman primates remains uncertain, and their mechanisms of action on placental tissues are not fully understood. Lastly, little is known about the long-term effects of placenta-targeted strategies on the health of the adult offspring^[162]^.

Significant challenges remain in developing and implementing MSC-EVs as pregnancy therapeutics. Nonetheless, placental MSC-EVs offer notable advantages, including immunomodulatory properties, natural tropism for the placenta, and a rich cargo of miRNAs, growth factors, and cytokines capable of activating multiple cellular repair pathways through paracrine signalling. These characteristics potentially make EVs superior to synthetic nanoparticle-based therapeutics, such as liposomes, which must be bioengineered to achieve placental targeting, carry only a limited set of biomolecules or drugs, and may trigger immune responses. Therefore, natural therapeutic EVs could play a key role in treating pregnancy disorders such as PE and FGR, both of which have multifactorial aetiologies and may require modulation of multiple biological pathways to repair placental damage. However, synthetic nanoparticles do have some advantages over EVs: they are less complex and variable, easier to manufacture, and exhibit longer circulation times. In the future, the most effective approach for placental therapy may involve bioengineered EVs capable of delivering therapeutic drugs or hybrid EV-synthetic nanoparticles (HEVs) that combine the benefits of both systems^[165]^.

## CONCLUSION

EVs hold great potential for the development of therapeutics targeting pregnancy disorders. EV research has deepened our understanding of their roles in normal placental development, as well as in the pathogenesis and pathophysiology of clinically significant pregnancy disorders. Abnormal MSC-EVs isolated from women with such disorders can be used to damage human or animal cells in *ex vivo* models or injected into animals to create *in vivo* models of human pregnancy diseases. These models support the testing of therapeutic MSC-EVs derived from women with healthy pregnancies, as well as therapeutic miRNAs, MSC-EV-associated microRNA biomarkers, and candidate drugs. NAMs employing “humanised models” are likely to generate advanced and comprehensive models of pregnancy pathologies, which will inform the rational development of MSC-EV-based therapies for future clinical trials. Finally, bioengineering will likely be required to improve the specificity and efficacy of MSC-EVs, enabling them to target the placenta precisely and perform their paracrine repair functions. The broader field of EV-based therapeutics is advancing rapidly, and many of the current barriers outlined in this review are expected to be overcome in the near future.

## DECLARATIONS

### Copyright

© The Author(s) 2025.
